# S2RSLDB: a comprehensive manually curated, internet-accessible database of the sigma-2 receptor selective ligands

**DOI:** 10.1186/s13321-017-0191-5

**Published:** 2017-01-21

**Authors:** Giovanni Nastasi, Carla Miceli, Valeria Pittalà, Maria N. Modica, Orazio Prezzavento, Giuseppe Romeo, Antonio Rescifina, Agostino Marrazzo, Emanuele Amata

**Affiliations:** Department of Drug Sciences, Medicinal Chemistry Section, University of Catania, Viale A. Doria 6, 95125 Catania, Italy

**Keywords:** Sigma receptor, Sigma-2 receptor, Online ligand database, Structure search, S2RSLDB, 2D plot, 3D plot, Drug design, Central nervous system multiparameter optimization, Lipinski’s rule of five

## Abstract

**Background:**

Sigma (σ) receptors are accepted as a particular receptor class consisting of two subtypes: sigma-1 (σ_1_) and sigma-2 (σ_2_). The two receptor subtypes have specific drug actions, pharmacological profiles and molecular characteristics. The σ_2_ receptor is overexpressed in several tumor cell lines, and its ligands are currently under investigation for their role in tumor diagnosis and treatment. The σ_2_ receptor structure has not been disclosed, and researchers rely on σ_2_ receptor radioligand binding assay to understand the receptor’s pharmacological behavior and design new lead compounds.

**Description:**

Here we present the sigma-2 Receptor Selective Ligands Database (S2RSLDB) a manually curated database of the σ_2_ receptor selective ligands containing more than 650 compounds. The database is built with chemical structure information, radioligand binding affinity data, computed physicochemical properties, and experimental radioligand binding procedures. The S2RSLDB is freely available online without account login and having a powerful search engine the user may build complex queries, sort tabulated results, generate color coded 2D and 3D graphs and download the data for additional screening.

**Conclusion:**

The collection here reported is extremely useful for the development of new ligands endowed of σ_2_ receptor affinity, selectivity, and appropriate physicochemical properties. The database will be updated yearly and in the near future, an online submission form will be available to help with keeping the database widely spread in the research community and continually updated. The database is available at http://www.researchdsf.unict.it/S2RSLDB.

**Graphical abstract:**

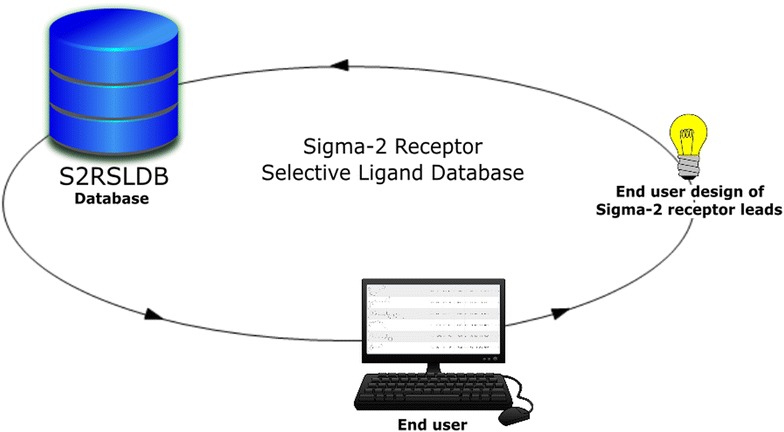

## Background

Sigma (σ) receptors are accepted as a particular receptor class consisting of two subtypes: sigma-1 (σ_1_) and sigma-2 (σ_2_). They are distinguished by molecular weight (MW), drug actions, pharmacological profiles and molecular characteristics [1, 2]. The σ_1_ receptor has a MW of 25.3 kDa and was first cloned from guinea pig liver (UniProtID Q60492, Gene names SIGMAR1, CHEMBL4153) in 1996 [3, 4], and afterwards from human placental choriocarcinoma cell (UniProtID Q99720, Gene names SIGMAR1, CHEMBL287) [5]. In addition, the σ_1_ receptor was also cloned by other organisms, like mouse (UniProtID O55242, CHEMBL3465), rat (UniProtID Q9R0C9, CHEMBL3602), brushtail possum (UniProtID Q5PXE2), ermine (UniProtID Q5PXE3), bovine (UniProtID Q58DH7), chicken (UniProtID Q5ZL84), and zebrafish (UniProtID Q7ZWG9). Recently the crystal structures of the human σ_1_ receptor in complex with two ligands, has been reported (PDB ID 5HK1 and 5HK2) [3, 6].

The σ_2_ receptor has not yet been cloned or crystallized and the knowledge about this receptor has mostly been generated through in vitro receptor radioligand binding studies [7, 8]. As reported the σ_2_ receptor has a MW between 18 and 21 kDa [9, 10]. It has been postulated that the σ_2_ receptor binding site may be located in the progesterone receptor membrane component 1 (PGRMC1), even if its MW (25 kDa) is different from that of σ_2_ receptor [10, 11].

The σ_1_ receptor is involved in aging and various diseases, like schizophrenia, depression, Alzheimer’s disease and ischemia. The σ_1_ receptor agonists have showed neuroprotective, anti-amnestic and antidepressant effects [12–14]. Conversely, σ_1_ receptor antagonists are considered antiproliferative, antiangiogenic and to have modulatory effects on opioid analgesia [15–17]. Some studies suggested that σ_1_ receptor is involved in modulating the synthesis and release of dopamine and also to act as a molecular chaperone at the mitochondrion-associated endoplasmic reticulum membrane (MAM) where it regulates calcium signaling between the two organelles [4, 18–20].

Despite the lack of structural information, the σ_2_ receptor has gained remarkable attention due to its involvement in several human diseases, including but not limited to depression, anxiety and cancer diagnosis and treatment [21–23]. The σ_2_ receptor ligands determine tumor cell death through apoptotic and non-apoptotic pathways, although their mechanisms of action have not been fully elucidated [24, 25]. In addition, the overexpression of σ_2_ receptor in several tumor cell lines is noteworthy [26–28]. The σ_2_ receptor is expressed about tenfold more in proliferating tumor cells compared with quiescent tumor cells, keeping the σ_2_ receptor ligands highly indicate for ligand-targeted cancer therapeutic strategies and as imaging agents [23, 29–32]. This peculiarity has been used for the development of σ_2_ receptor selective ligands as Positron Emission Tomography (PET) imaging tools. [^18^F]ISO-1, a promising PET ligand targeting σ_2_ receptor, has been evaluated in clinical trial for the assessment of cellular proliferation in tumors by PET and three additional phase I clinical trials on this compound are actually ongoing [33–36]. These differences in the pharmacological profiles of the σ receptor subtypes, prompt to a continue research of ligands that selectively target each of them. However, whilst several ligands selectively bind to the σ_1_ receptor or indistinctly to the two receptor subtypes, the development of compound endowed with high selectivity for the σ_2_ receptor has been challenging and in some cases occurred through an accidental discovery [23].

Due to the lack of structural information about the σ_2_ receptor and its growing implication in cancer diagnosis and treatment, a thorough and in-depth collection of the selective σ_2_ receptor ligands could result in a helpful tool for drug discovery. Herein, an online ligand database named sigma-2 Receptor Selective Ligands Database (S2RSLDB) based on 2D structural information, computed physicochemical properties, pharmacological properties together with the experimental procedure protocols, retrieved from the literature, has been built and resulted in more than 650 compounds. The database contains all the ligands that selectively bind the σ_2_ receptor (*i.e. K*_i_ σ_1_/σ_2_ > 1). The S2RSLDB is freely available online without account login and having a powerful search engine the user may build complex queries, sort tabulated results, generate color coded 2D and 3D graphs and download the data for additional offline screening.

The collection here reported is extremely useful for the development of ligands endowed of σ_2_ receptor affinity, selectivity, and appropriate physicochemical properties. To the best of our knowledge, there is not any online database reporting such complete compounds map for this receptor. Moreover, in most cases these do not allow a comparison between the compound’s features and a complete and correct set of compounds is difficult to be returned. The database will be updated yearly and in the near future, an online submission form will be available to help with keeping the database widely spread in the research community and continually updated.

## Description and utility

Compound information was manually retrieved from the literature, including journal articles and patents, which were selected using major databases like Pubmed [37], SciFinder [38], and Google. The Binding Database [39], ChEMBL (v21) [40], PubChem [41], PDBbinding [42], ChemSpider [43], as well as other online ligand databases, were also checked for completeness. For each literature source, a curator manually constructed the 2D chemical structures using Marvin Sketch (v14.9.1.0) [44] and converted them into SMILES strings using JChem for Excel (v14.9.100.809) [44]. For few publications, the Optical Structure Recognition Application (OSRA) software (v2.0.1) was employed to generate SMILES [45]. A SMILES file was generated and then converted them into structures using JChem for excel (v14.9.100.809) [44]. A second curator visually inspected for common structure mistakes. Once a full list of compounds was available, compound structures were verified in SciFinder [38], patent and journal articles by SMILES or 2D structure visual inspection. Finally, Open Babel (v2016-01) was used to create InChI strings that in turn were used to remove duplicates from the S2RSLDB [46].

Other information taken from the literature includes the radioligand binding affinity values (*K*_i_ or IC_50_ expressed in nM or otherwise converted in nM), the experimental procedure protocols [47], the reference article compound key and/or preferred name (*e.g.* CM-361; 9f), and the compound formulation (free base or salt) used for the displacement binding assay. Those compounds presenting numerous binding affinity values, resulted from different experimental conditions, have been associated with multiple experimental data. Indeed, for several compounds, significant variations in binding assay output values have been encountered and for others an inversion in the selectivity ratio (*K*_i_ σ_1_/σ_2_ < 1) has been observed.

**Fig. 1:**
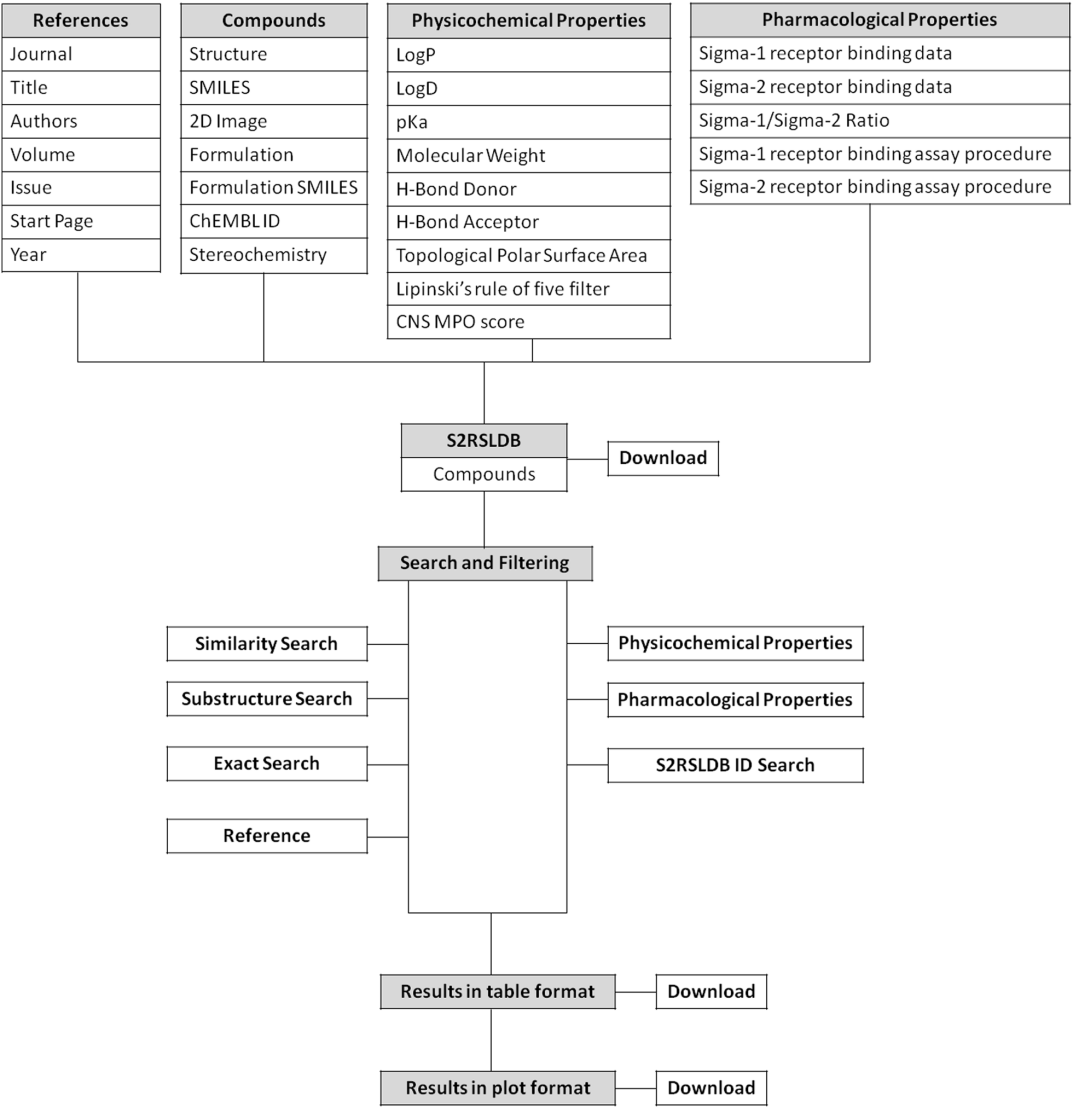
Schematic representation of the S2RSLDB configuration

S2RSLDB is available online at http://www.researchdsf.unict.it/S2RSLDB. The database is implemented in MySQL (v5.1.73) with Apache (v2.4.20) as the web server. For chemical calculation and structure drawing Open Babel (v2016-01), Pybel (v2.3.1) and JSME Applet (v2015-12-06) are incorporated [46, 48, 49]. Other functions are made available with Python scripts and background program. The data are stored in MySQL (v5.1.73) database. The website is built in HTML, JavaScript, CSS and PHP. Compound images have been generated through Indigo Toolkit (v1.2.1) [50]. The types of data stored and the database structure are illustrated in Fig. 1.

The S2RSLDB has a powerful and intuitive web interface. The search page is divided into five sections: structure, computed physicochemical properties, pharmacological properties, reference search, and S2RSLDB-ID. Structure search may be performed by either drawing a molecule using the JSME Molecular Editor or entering a SMILES string as query input. Within the structure search, similarity, substructure, and exact search can be run. Similarity search employs the FP2 fingerprint, substructure search is performed via SMARTS while exact search is done through InChI strings correlation [51–53]. By default, the website is set to perform similarity search with a Tanimoto coefficient cutoff of zero [51]. With this setting, all the compounds in the database are returned to the query, and the result webpage will give a tabulation of the compounds sorted by Tanimoto coefficient. All the aforementioned functionalities are done via Pybel [48].

**Fig. 2:**
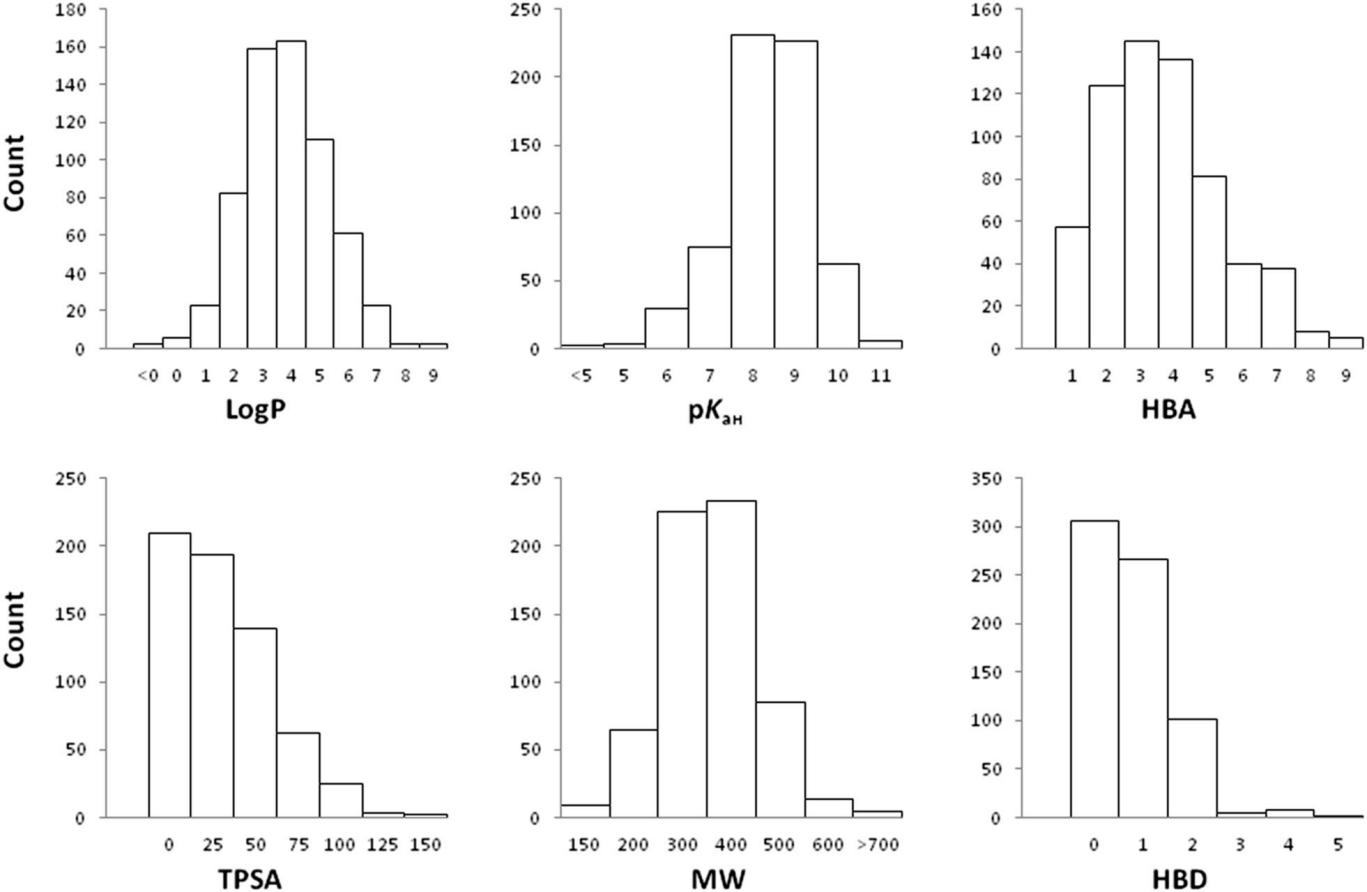
Distribution of the physicochemical properties of the compounds in the database

**Fig. 3:**
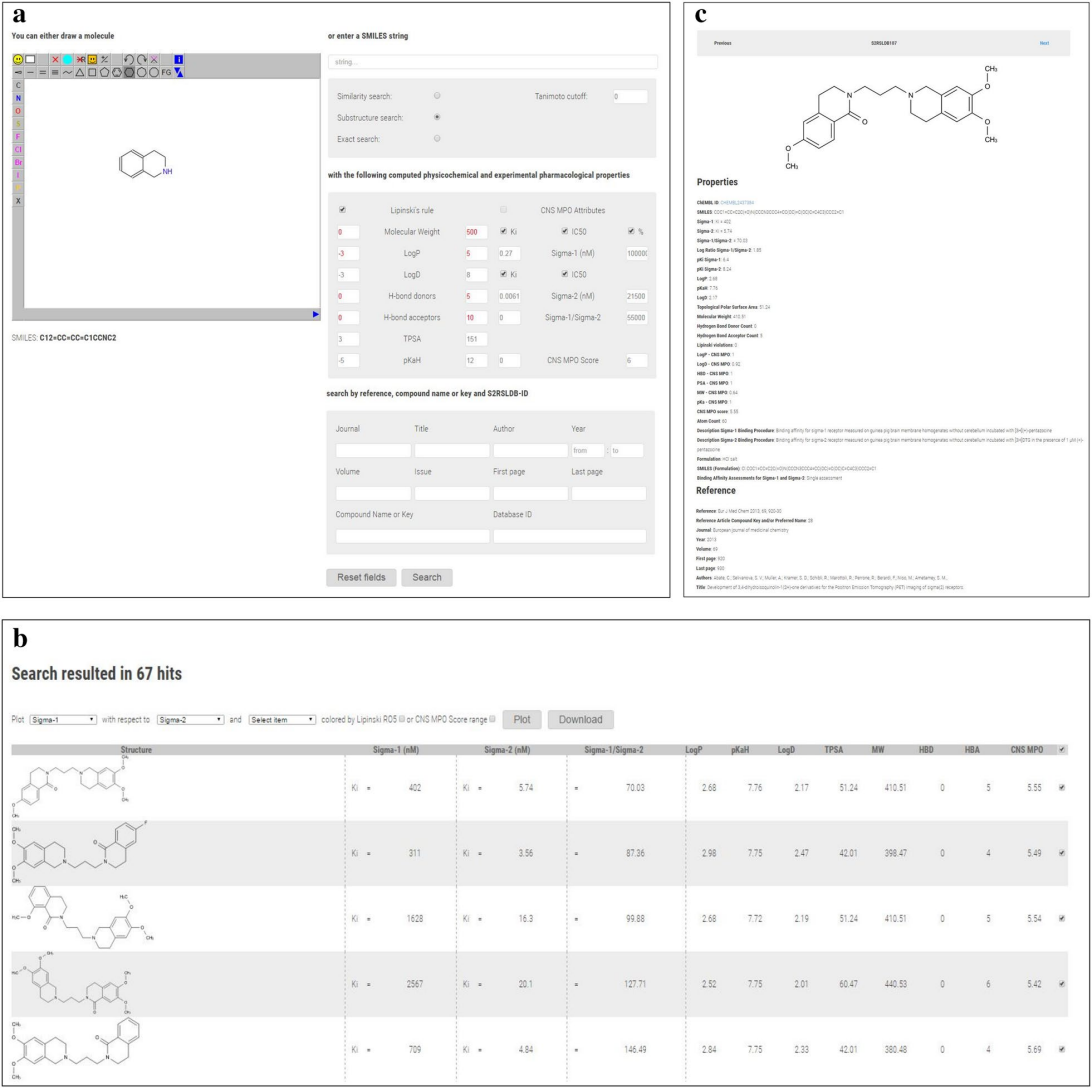
Screenshots of the compound search page set with 1,2,3,4-tetrahydroisoquinoline substructure search and Lipinski’s rule of five filter (**a**), tabulated results page (**b**), and compound summary page (**c**)

Physicochemical properties search contains selected molecular properties which were calculated for all compounds using ChemAxon’s calculator cxcalc (v6.1.3) [44]. These descriptors include MW, octanol–water partition coefficient (LogP), H-bond donors (HBD), H-bond acceptors (HBA), which allow the creation of the Lipinski’s rule of five filter [54]. A Lipinski’s rule of five filter checkbox has been incorporated for helping the end user in automatically set the filter cutoff. Other descriptors include LogD [pH 7.4], topological polar surface area (TPSA), atom count, and p*K*_aH_ (calculated for the most basic center). A histogram distribution of these computed physicochemical properties for the compounds in the database is presented in Fig. 2. For alignment of the key druglike attributes, the central nervous system multiparameter optimization (CNS MPO) score has been calculated for each compound and a CNS MPO score box has been included in the search page utility interface [55]. This score (0–6 range) is the sum of a set of six normalized (0 to 1 range) physicochemical parameters: logP, logD, MW, TPSA, HBD, and pK_aH_ and may help the user to predict each compound’s likelihood of CNS activity and overall better druglike properties [55, 56]. Compounds having a CNS MPO ≥ 4 show better druglike properties and are predicted to be centrally acting. [55, 56]. Furthermore, by activating the CNS MPO attributes input checkbox the six physicochemical properties boxes composing the score, will be returned in the normalized fashion according to the CNS MPO algorithms. For each computed physicochemical property, cutoff or range can be specified by changing the number in the search webpage boxes. In Fig. 3a, Lipinski’s rule of five filter was applied together with a substructure search of 1,2,3,4-tetrahydroisoquinoline and this returned 67 hit compounds subjected to the rules. Once the search function is launched, matching queries are returned on a result webpage in tabulated format displaying main pharmacological and computed physicochemical properties together with the CNS MPO score as well as the 2D image of the compounds (Fig. 3b). On condition that the CNS MPO input checkbox has been activated, the result webpage will show the six computed physicochemical properties composing the score normalized according to the CNS MPO algorithms. Each compound may thus be opened as a summary page (Fig. 3c).

Similar filtering options are available for the pharmacological properties search. This search function also includes which type of standard constant to be displayed (IC_50_, *K*_i_, and % of inhibition) for both receptors and the σ_1_/σ_2_ ratio (*i.e.* selectivity). The reference filter search function allows the end user to customize results based on journal name, article title, author name, volume, issue, page and year of publication. Finally, to help the user in retrieving a specific compound from the database S2RSLDB-ID and the reference article compound key and/or preferred name (as reported in the literature source) searches have been embedded.

The user may build complex queries performing searches on a number of fields simultaneously. All the numerical fields in the tabulated results webpage can be sorted, allowing for an enhanced data analysis. Tabulated result page sorting is accomplished with Sorttable (v2) [57]. Compounds can be selected for download in excel binary file format (xls). All the entries in the page are selected by default and a check-all button lets the user select/deselect and then download all the compounds displayed. Navigating in the download tab, the full database may be downloaded in two different file formats: structure-data file (sdf) and xls.

**Fig. 4:**
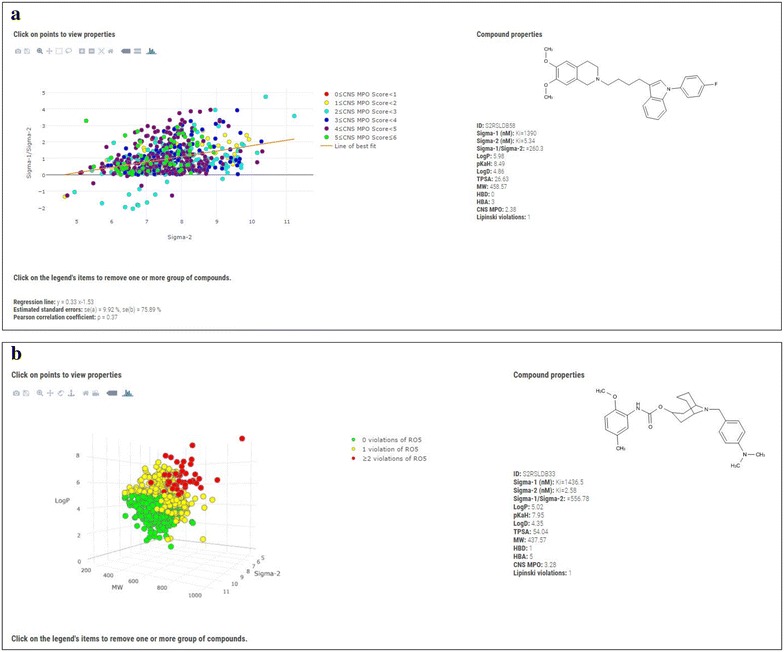
2D (**a**) and 3D (**b**) scatter distribution (website screenshots) of compound’s σ_2_ *K*_i_ versus σ_1_/σ_2_ ratio and of compound’s σ_2_ p*K*_i_ versus MW versus logP color coded by CNS MPO score (2D) and Lipinski’s rule of five filter (3D), and two compounds together with their property summaries

Of particular importance for the data set analysis is the opportunity to perform graphical analysis of the data set or subset. The user may build customized 2D and 3D scatter plots of the compounds selected from the tabulated results page, by defining the axes (eleven variables available: Sigma-1 *K*_i_, sigma-2 *K*_i_, sigma-1/Sigma-2 *K*_i_ ratio, Log*P*, p*K*_aH_, Log*D*, TPSA, MW, HBD, HBA, and CNS MPO) that in turn will compose the Cartesian coordinates of the 2D or 3D graph. A webpage will give the scatter plot for the defined axes. A simple linear regression model has also been added to the 2D graph and this feature should let the user to recognize pattern in the compound properties behaviour. Being x and y the two variables to be plotted, the fitted regression line has the following equation: y = ax + b, where a is the slope and b is the intercept of the estimated line. Therefore we estimated the standard error (se) for a and b, indicated on the database 2D plot webpage with se(a) and se(b). Finally, the Pearson correlation coefficient ρ is given in order to appreciate the data fitting [58]. The aforementioned functionalities are run with background software. The plots dots may also be color coded based on Lipinski’s rule of five violations or CNS MPO score range. A three or six color code can been applied as a function of the Lipinski’s rule of five violations or CNS MPO score range, respectively. Moreover, in the 2D and 3D plot webpage, an interactive box has been embedded and this feature allows the user to remove from the plot group of compounds with specific Lipinski’s rule of five violations or CNS MPO score range. The graphs are interactive and moving the cursor over a point, will show the coordinates as a tooltip, whilst by clicking a point of the graph, the 2D image of the selected compound and a properties summary will be returned in a quadrant. The plotting is made available by plotly.js (v1.10.2) [59] which is embedded in the website. Since the pharmacological properties (experimental radioligand binding assay) are variables with a wide numerical distribution, for plotting purposes, we have reported them as −log of the *K*_i_ or IC_50_ (i.e. as p*K*_i_ or pIC_50_). For the same reason, the σ_1_/σ_2_ ratio is showed in logarithm scale (log σ_1_/σ_2_ ratio). This last function may be used prospectively at the design stage to accelerate the identification of σ_2_ receptor ligands with increased probability of selectivity over σ_1_ receptor. The plots may be downloaded as image files for further offline purposes. Figure 4 shows the 2D (a) and 3D (b) scatter distribution (website screenshots) of compound’s σ_2_ *K*_i_ versus σ_1_/σ_2_ ratio and of compound’s σ_2_ p*K*_i_ versus MW versus logP color coded by CNS MPO score (2D) and Lipinski’s rule of five filter (3D), and two compounds together with their property summaries.

## Conclusion

The σ_2_ receptor structure has not yet been disclosed, and radioligand binding assay is primarily used to understand the receptor’s pharmacological behavior and design new lead compounds. With this in mind, here we present a comprehensive, manually curated, database of the σ_2_ receptor selective ligands containing more than 650 compounds, built with chemical structure information, radioligand binding affinity data, computed physicochemical properties, and experimental binding protocols. The reported data have been manually retrieved from the literature thus keeping this database highly reliable. Each compound in the database has the reference source. The S2RSLDB is freely available online without account login and having a powerful search engine the user may build complex queries, sort tabulated results, generate 2D and 3D color coded graphs and download the data for additional offline screening. The collection here reported is extremely useful for the development of new ligands endowed of σ_2_ receptor affinity, selectivity, and appropriate physicochemical properties.

## Abbreviations

CNS MPO: central nervous system multiparameter optimization
TPSA: topological polar surface area
MW: molecular weight
PET: positron emission tomography
SMILES: simplified molecular-input line-entry system
SMARTS: SMiles ARbitrary target specification
InChI: IUPAC international chemical identifier
HTML: hypertext markup language
CSS: cascading style sheets
JSME: JavaScript molecule editor
2D: two-dimensional
3D: three-dimensional
S2RSLDB: sigma-2 receptor selective ligands database
